# Inherited Cardiac Arrhythmia Syndromes: Focus on Molecular Mechanisms Underlying TRPM4 Channelopathies

**DOI:** 10.1155/2020/6615038

**Published:** 2020-12-16

**Authors:** Mohamed-Yassine Amarouch, Jaouad El Hilaly

**Affiliations:** ^1^R.N.E Laboratory, Multidisciplinary Faculty of Taza, University of Sidi Mohamed Ben Abdellah, Fez, Morocco; ^2^Department of Biology and Earth Sciences, Regional Center for Education Careers and Training, Fez, Morocco

## Abstract

The Transient Receptor Potential Melastatin 4 (TRPM4) is a transmembrane N-glycosylated ion channel that belongs to the large family of TRP proteins. It has an equal permeability to Na^+^ and K^+^ and is activated via an increase of the intracellular calcium concentration and membrane depolarization. Due to its wide distribution, TRPM4 dysfunction has been linked with several pathophysiological processes, including inherited cardiac arrhythmias. Many pathogenic variants of the *TRPM4* gene have been identified in patients with different forms of cardiac disorders such as conduction defects, Brugada syndrome, and congenital long QT syndrome. At the cellular level, these variants induce either gain- or loss-of-function of TRPM4 channels for similar clinical phenotypes. However, the molecular mechanisms associating these functional alterations to the clinical phenotypes remain poorly understood. The main objective of this article is to review the major cardiac TRPM4 channelopathies and recent advances regarding their genetic background and the underlying molecular mechanisms.

## 1. Introduction

The Transient Receptor Potential Melastatin 4 (TRPM4) is a homotetrameric N-glycosylated ion channel that belongs to the large family of TRP proteins [1–5]. It is an intracellular calcium-activated nonselective channel permeable to monovalent cations with the following selectivity Na^+^≈K^+^>Cs^+^>Li^+^. TRPM4 shows an equal permeability to Na^+^ and K^+^ ions, and it is activated via an increase of the intracellular calcium concentration and membrane depolarization [6, 7]. However, it does not show any permeability to the Ca^2+^. When TRPM4 is activated, it mediates sodium influx into the cell at negative potentials inducing a membrane depolarization. In contrast, at positive potentials, an efflux of potassium leading to membrane repolarization is observed [6, 8].

TRPM4 is involved in the regulation of several physiological processes, including cardiac excitability and automaticity [9–11], insulin secretion by pancreatic cells [12], and immune cell activity [13]. In this sense, several studies have shown the presence of TRPM4-related current, NSC_Ca_, in the cardiovascular system, especially in cardiac cells of the conduction pathways, in cerebral arteries, as well as in immune cells [13–18].

TRPM4 displays a widespread expression in various cells and tissues. Therefore, TRPM4 has been linked with several human diseases including neurological and cardiac disorders. In the heart, TRPM4 has been associated with different forms of inherited cardiac arrhythmias through the identification of many pathogenic genetic variants in the affected patients [19–23]. The identified mutations induce either gain- or loss-of-function of TRPM4 channels. However, the molecular mechanisms of this association are not yet clearly unveiled.

The present review discusses the pathophysiological implications of TRPM4 dysfunction in inherited cardiac arrhythmias, especially inherited cardiac conduction disorders, Brugada syndrome, and the congenital long QT syndrome.

## 2. TRPM4 Channels: From Biophysics to Physiology

### 2.1. Structure and Function of TRPM4 Channels

**Figure 1:**
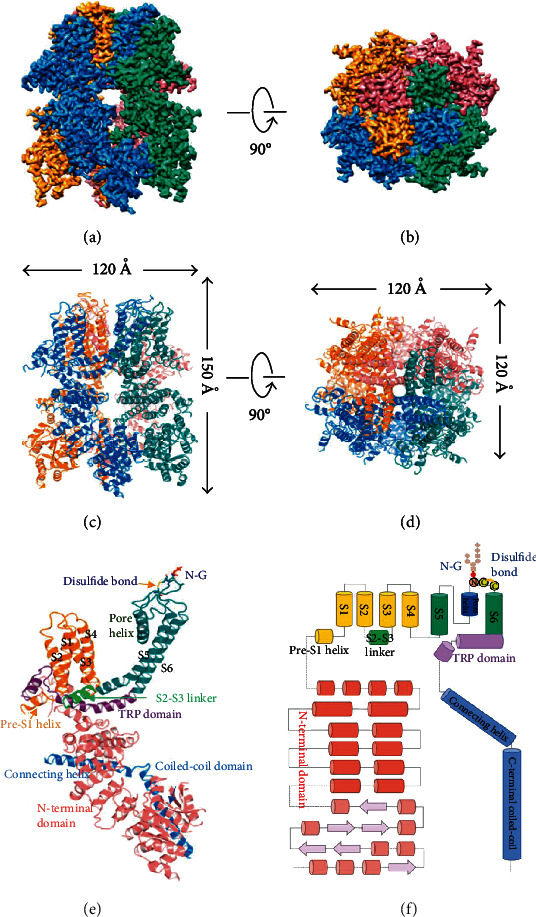
Overall structure of human full-length TRPM4 in the apo state. (a, b) Side and top views of the cryo-EM reconstruction density map of human TRPM4 at 3.7 Å overall resolution. (c, d) Ribbon diagrams representing the same orientation and colors with the channel's dimensions indicated. (e) Structural details of a single human TRPM4 subunit. (f) Linear diagram depicting the major structural domains, color coded to match the ribbon diagram. The N-linked N992 glycosylation site (N-G) and the Cys993-Cys1011 disulfide bond are indicated; reprinted from [2].

Like other TRP channels, TRPM4 contains multiple transmembrane and cytosolic domains. Several studies have described the three-dimensional structure of TRPM4 as tetrameric structure. Each monomer contains four transmembrane helices (S1–S4), a pore domain located between the S5 and S6 helices, and cytosolic N and C termini (Figure 1) [1–3, 5].

In contrast to other voltage-gated ion channels, the 3D structure of TRMP4 does not show the four arginine residues that define the voltage-sensor domain. Two arginine residues (R892 and R905) have been found at either end of S4 with a minor contribution to the voltage sensitivity of TRMP4 [2]. Moreover, an extracellular glycosylation site at the residue N992 was identified on each monomer. These findings are in line with Abriel's group study. These authors have demonstrated that the abolishment of N-linked glycosylation of TRPM4 channel (by substituting the N992 to a Glutamine) decreases the current density without affecting the number of the mutated TRMP4 channels at the plasma membrane [4].

At the functional level, the expression of TRPM4 channels in HEK293 cells induces an ionic current characterized by a linear unitary current-voltage relationship and a conductance between 20 and 25 pS [6]. In addition, an endogenous TRPM4-like conductance has been recorded in these cells [24].

TRPM4 is regulated by multiple factors such as phosphatidylinositol 4,5-bisphosphate (PIP_2_), hydrogen peroxide, PKC, ATP, and calmodulin [25–30]. The PIP_2_ has been described to be one of the major modulators of TRPM4 channel. It implicates the C-terminal pleckstrin homology domain of TRMP4 in the PIP_2_ sensing. On the functional level, PIP_2_ rescues TRPM4 from desensitization, increases its sensitivity to calcium, and shifts its voltage dependence of activation towards negative potentials [27, 30]. In addition, TRPM4 interacts with different types of proteins including ion channel subunits. Accordingly, Park et al. have shown, using yeast two-hybrid screening and immunoprecipitation techniques, a protein-protein interaction between TRPM4 and TRPC3 channels [31]. Consequently, the activation of TRPM4 inhibits TRPC3-mediated currents by more than 90% [31]. Another example of TRPM4 protein-protein interactions is illustrated by its association with Sulfonylurea Receptor 1 (SUR1), 14-3-3*γ*, and protein tyrosine phosphatase nonreceptor type 6 (PTPN6). The coexpression of TRPM4 with SUR1 was shown to increase its affinity for calmodulin as well as its sensitivity to intracellular calcium [32]. However, the interaction with 14-3-3*γ* and PTPN6 mediates the membrane targeting of TRPM4. In fact, 14-3-3*γ* and PTPN6 depletion disrupts trafficking of TRPM4 channels to the plasma membrane and subsequently abolishes its activity [33, 34].

### 2.2. TRPM4 in the Heart

TRPM4 channel is expressed in different mammalian cardiac cells. Its mRNA, protein, or related current is detected in atrial and ventricular cardiomyocytes, sinoatrial node, conductive tissue, and atrial and ventricular fibroblasts [9, 11, 15, 17, 18, 23, 35–39].

The atrial function of TRPM4 channels was explored by Simard group [11]. They have compared the electrical activities of atrial cardiomyocytes from TRPM4^+/+^ and TRPM4^−/−^ mice [11]. The obtained single channel recordings have revealed a typical TRPM4 current in WT mice, whereas no activity was detected from TRPM4^−/−^. Moreover, by evaluating the impact of TRPM4 gene invalidation on the atrial action potential (AP), the authors have suggested that TRPM4 is involved in the repolarization phase of the atrial AP [11]. These results were supported by the effect of TRPM4 inhibitors on the shape of the atrial AP. Indeed, flufenamic acid and 9-phenonthrol have shown a concentration-dependent decrease in AP duration, and this effect is abolished in the TRPM4^−/−^ mice [11]. Accordingly, these results were corroborated by Demion et al.'s study reporting a reduced atrial AP in TRPM4^−/−^ mice [40].

More recently, Son et al. have stated that TRPM4 channels could be indirectly activated by shear stress in atrial myocytes [41]. The activated TRPM4 current seems to be the major component of the shear-sensitive current. Besides, the authors have emphasized that calcium release through type 2 inositol 1,4,5-trisphosphate receptors (IP3R2) plays a key role in this Ca^2+^-dependent activation of TRPM4 [41].

In addition to its atrial expression, TRPM4 activity was also detected in sinoatrial node (SAN) cells [15]. Indeed, a calcium-activated nonselective cation conductance was recorded using the inside-out configuration of the patch clamp technique in freshly isolated SAN cells from adult mice. This conductance presents several biophysical characteristics of TRPM4 channels such as a conductance of 20.9 ± 0.5 pS and equal permeability to Na^+^ and K^+^. These results are sustained by the detection of TRPM4 expression on SAN cells by RT-PCR and Western blot techniques [15]. Regarding the physiological relevance of these findings, Hof and colleagues have investigated the implication of TRPM4 channels on the sinus rhythm function in mice, rat, and rabbit [10]. They have studied the impact of 9-phenonthrol on spontaneous APs from right atria and demonstrated that this compound produces a dose-dependent reduction in AP rate. This effect was higher at low rates, mediated by a reduction in diastolic depolarization slope, and absent in TRPM4^–/–^ mice [10].

On the other hand, Guinamard's group has investigated the functional implication of TRPM4 by exploring the electrical activity of rabbit Purkinje fibers in the presence and absence of 9-phenanthrol [9], which result in decreasing the Purkinje AP duration. Furthermore, inside-out and whole-cell patch clamp recordings have revealed a TRPM4-like single channel activity and a transient inward 9-phenanthrol-sensitive current, respectively [9]. These findings have shown that TRPM4 affects the electrical activity of cardiac Purkinje fibers and thus could influence cardiac conduction.

Finally, the functional role of TRPM4 in ventricles is still on debate, albeit most of the studies have found its weak expression [22, 36, 42, 43]. Mathar and colleagues showed a shortened ventricular AP duration in TRPM4^–/–^ mice, while Demion and colleagues did not report any modification of the ventricular AP shape [18, 40]. However, the comparison between the two studies should be taken with caution, because their experimental conditions were different. Mathar and colleagues have performed microelectrode AP measurements in tissue strips, whereas Demion and colleagues performed isolated cellular AP recordings. Moreover, the genetic background of the used TRPM4 knockout mice was derived from different mouse strains.

## 3. Inherited Cardiac Disorders of TRPM4 Channels

Cardiac channelopathies are genetic disorders triggered by ion channel dysfunction, which is the principal pathophysiological mechanism underlying various inherited forms of cardiac arrhythmias. In this sense, a large number of mutations in genes encoding ion channels and their regulatory proteins have been associated with an increasingly wide range of inherited cardiac disorders. Examples of genetic disorders include cardiac conduction disorders, Brugada Syndrome, and long QT syndrome. Many of these disorders have been linked with TRPM4 dysfunction.

### 3.1. Cardiac Conduction Disorders

Cardiac conduction disorders (CCD) are potentially life-threatening diseases characterized by an impaired cardiac conduction at different heart regions such as atrium, atrioventricular node, and ventricles. Electrocardiogram (ECG) of the affected patients may show a prolonged P-wave or PR interval, QRS widening, bundle branch blocks, and atrioventricular block. CCD could be attributed to a variety of factors including injury, structural heart diseases, or be found isolated without any structural abnormalities [44–46]

In this context, molecular genetic investigations of idiopathic CCD cases have allowed identifying many pathogenic variants in genes encoding transcription factors, transmembrane transporters, and structural proteins [44–46]. Within these genes, a growing number of TRPM4 variants have been associated with different forms of cardiac conduction disease [19, 21–23, 42, 47, 48]. The first TRPM4 mutant, TRPM4-p.E7K, was identified by Kruse et al. in a large South African Afrikaner pedigree with an autosomal-dominant form of progressive familial heart block type I (PFHB1) [22]. The electrophysiological investigations of the TRPM4-p.E7K mutant revealed an increased whole-cell current without any modification of the unitary biophysical properties, as compared to WT channel. This effect was attributed to an attenuated deSUMOylation process, which represents a reversible posttranslational mechanism modulating the activity, stability, or localization of intracellular proteins. In this light, the resulting constitutive SUMOylation of the p.TRPM4-E7K mutant impairs endocytosis and stabilizes this channel at the cellular surface [22]. Similarly, three other TRPM4 mutants (p.R164W, p.A432T, and p.G844D) were then identified in French and Lebanese families with autosomal dominant isolated cardiac conduction blocks [23]. The electrophysiological characterization of these mutants revealed a gain-of-function effect, which was proposed to be the results of an impaired endocytosis and deregulation of TRPM4 SUMOylation [23].

Daumy and colleagues have identified an additional TRPM4 variant in patients harboring PFHB1. The p.TRPM4-I376T variant was identified in a large French 4-generation pedigree with PFHB1. Expression and functional analyses of this variant were performed in HEK293 cells using whole-cell patch clamp, Western blotting, and cell surface biotinylation techniques. In the presence of the p.I376T variant, the authors reported an increase of the current density that was associated with an increased expression of TRPM4 channel at the plasma membrane [19].

Later, the genetic screening of 91 patients with childhood atrioventricular block allowed the identification of five rare TRPM4 variants (p.D198G, p.A432T/G582S, p.T677I, and p.V921I) [47]. The variants p.D198G, p.T677I, and p.V921I do not induce any significant changes of TRPM4 expression and function. The p.A432T and p.A432T/G582S variants showed a decrease of TRPM4 expression at the cell surface. However, the electrophysiological characterization of these variants revealed a decrease in the current density of TRPM4-p.A432T channel and no significant changes for TRPM4-p.A432T/G582S [47]. The observed loss-of-function was proposed to be related to a folding and trafficking defect. The expression and function of TRPM4-p.A432T and TRPM4-p.A432T/G582S mutants were shown to be partially rescued by incubation at low temperature [47]. Regarding the TRPM4-p.G582S mutant, an increased expression and function were observed [47]. However, the present study did not reveal any evidence of direct SUMOylation of either the TRPM4-WT or p.G582S variant, as it was reported by two previous studies [22, 23]. There is also a disagreement between Liu et al. and Syam et al., regarding the functional effect of the p.A432T variant. Liu and colleagues showed a gain-of-function effect of the p.A432T variant, while Syam and colleagues reported a loss-of-function effect. Xian's group have more deeply investigated the biophysical properties the TRPM4-pA432T mutant [48]. The authors have optimized assays mimicking the rapid and dynamic increases of intracellular Ca^2+^ in cardiac cells, which lead to kinetically characterize TRPM4 current while avoiding a prolonged exposure to high Ca^2+^ concentrations. In HEK293 cells expressing WT or TRPM4-p.A432T channels, the biophysical properties of TRPM4 channels were investigated using fast photolytic uncaging of caged Ca^2+^. TRPM4 currents were activated by trains of UV showing a gain-of-function in the mutated condition. Moreover, the kinetic analysis revealed a slower deactivation for the TRPM4-p.A432T channels, which lead to a progressive current increase during repetitive human cardiac action potentials. Accordingly, this study suggested that the slow deactivation might affect the cardiac action potential shape and result in aberrant impulse propagation and distribution on the tissue level [48].

Finally, Bianchi and colleagues have found and characterized four novel TRPM4 variants in patients with complete heart block [49]. Biochemical and electrophysiological characterization of these variants revealed a decreased protein expression and function for p.A101T, p.S1044C, and p.A101T/P1204L compared to WT-TRPM4 channel, while p.Q854R showed an augmented TRPM4 current [49]. Then, to understand the cellular mechanism underling the loss- or gain-of-expression in TRPM4 variants, the protein turnover of these variants was investigated in HEK293 cells, using cycloheximide as an inhibitor of protein biosynthesis. The obtained results showed an increased degradation rate for the TRPM4 loss-of-expression variants, while the gain-of-expression variants showed a higher stability compared to the WT condition. Based on these findings, the authors concluded that the protein expression of the identified TRPM4 variants may result from an altered TRPM4 half-life compared to the WT channels [49].

Other TRPM4 variants (p.Q131H, p.Q293R, p.G582S, p.Y790H, p.K914X, and p.P970S) were identified in patients with right-bundle branch block and atrioventricular block. However, no biochemical or functional data was shown [50].

A list of the TRPM4 variants linked with cardiac conduction disorders is summarized in Table 1.

### 3.2. Brugada Syndrome

**Figure 2:**
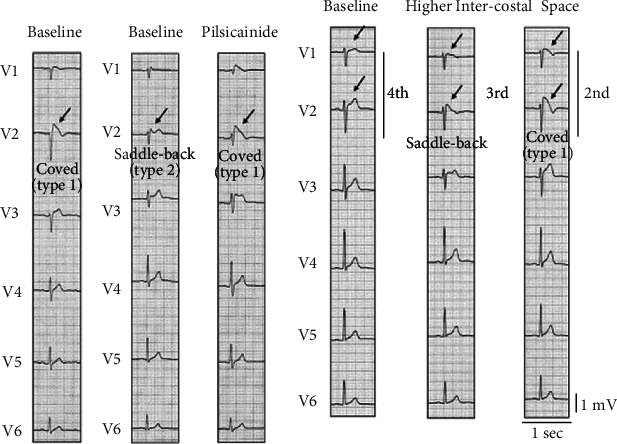
Brugada syndrome ECGs. (a) Spontaneous type 1 ST-segment elevation. (b) Unmasking of ST-segment elevation by a pharmacological sodium channel blocker, pilsicainide. Under baseline condition, type 2 ST-segment elevation is recorded in lead V2. Pilsicainide injection (30 mg) unmasks type 1 electrocardiogram (ECG) in lead V2. (c) Unmasking of type 1 ECG by recordings of right precordial (V1–V2) leads at the third and second intercostal spaces. Adapted and reprinted with permission from [52].

Brugada syndrome (BrS) is a rare inherited cardiac channelopathy characterized by an ST-segment elevation in the right precordial leads on the ECG and an increased risk of sudden cardiac death (Figure 2). Three distinct ECG patterns have been associated with this syndrome. According to the 2017 expert consensus conference on J-wave syndromes, only a type 1 ST-segment elevation should be considered diagnostic of BrS. This ECG pattern is characterized by ST-segment elevation ≥ 2 mm (0.2 mV) in ≥1 right precordial leads positioned in the 4th, 3rd, or 2nd intercostal space (Figure 2) [51]. When a type 1 ST-segment elevation does not spontaneously occur, a pharmacological challenge may be performed to unmask this pattern (Figure 2). In this case, diagnosis of BrS should also require the consideration of other criteria such as a documented ventricular arrhythmia, a family history of sudden cardiac death with negative autopsy, and coved-type ECGs in family members [51].

Since its first description in 1992 [53], the genetics of BrS has been elusive in the majority of cases. This syndrome was initially considered as a Mendelian disease with an autosomal dominant inheritance mechanism and incomplete penetrance, with *SCN5A* as a pivotal gene involved in 15-30% of BrS cases [54–56]. Conversely, recent evidences suggest that the phenotypic expression of BrS could be the result of a cumulative effect of common genetic variations, which pleads in favor of an oligo- or polygenic model for this disease [57]. The genome-wide association study, published by Bezzina et al., has reported an association between multiple single nucleotide polymorphisms in three genes (*HEY2*, *SCN5A*, and *SCN10A*) and the ECG characteristics of affected patients. This indicates that common variants can modulate susceptibility to a rare arrhythmia disorder [57].

Regarding the electrophysiological mechanisms underlying BrS, the depolarization and repolarization hypotheses were proposed. The first one assumes that the conduction delay in the right ventricular outflow tract (RVOT) induces the development of the observed ST-segment elevation and arrhythmic manifestations of BrS, whereas the latter one presumes a high level of transmural dispersion of repolarization in the right ventricle, which facilitate to the development of phase 2 reentry.

The depolarization hypothesis is grounded on the presence of a conduction delay in the RVOT compared to the other regions of the right ventricle, which may constitute the arrhythmogenic area underlying the BrS [58–60]. These findings are consonant with several clinical investigations. This body of evidences posits a common location of the arrhythmogenic electrophysiological substrate in the right ventricle epicardium in symptomatic BrS patients presenting recurrent Ventricular Tachycardia/Ventricular Fibrillation (VT/VF) [58, 60–65]. In addition, catheter ablation of the arrhythmogenic substrate results in ECG normalization and VT/VF noninducibility, suggesting the effectiveness of this procedure to eliminate the arrhythmic consequences related to Brugada syndrome [60, 62].

On the other hand, the repolarization hypothesis is mainly founded on experimental evidences, which suggest the presence of a high level of the transmural dispersion of repolarization in the right ventricle. In this respect, experiments conducted on transmural ventricular wedges of canines revealed the creation of a transmural voltage gradient related to the loss of the action potential dome in the right ventricular epicardium in response to sodium channel inhibition [66, 67]. These observations were linked to the presence of a more prominent outward transient current (Ito) in the right ventricular epicardium. This makes the latter region prone to a successive action potential duration shortening and then leads to a coved-type ST-segment elevation in the ECG right precordial leads, phase 2 reentry, and triggers ventricular fibrillation.

For more in-depth overview of pathophysiological mechanisms of Brugada syndrome, these detailed reviews are worthy of note [54–56, 68].

As previously reported, several studies have linked many genetic variants of the TRPM4 gene to different forms of cardiac conduction diseases. These findings have led many authors to investigate the causative role of TRPM4 gene in BrS since this syndrome is usually associated with cardiac conduction disorders. In this line, Liu et al. have screened a cohort of 248 BrS cases with no *SCN5A* mutations, of which eleven TRPM4 variants were identified in 20 unrelated patients [69]. Among them, only four identified mutants (p.P779R, p.T873I, p.K914X, and p.L1075P) were considered as putative genetic BrS predisposing factors, and their electrophysiological and biochemical profiles were investigated. A significant decrease in current densities as well as a reduced channel expression was noticed in p.P779R and p.K914X mutants. Moreover, while the p.P779R mutant shows a “WT-like” single conductance and more depolarized voltage sensitivity, the p.K914X mutant does not exhibit any detectable unitary current [69]. Finally, no alteration of whole-cell current, single channel properties, and TRPM4 channel regulation was reported for the p.T873I and p.L1075P mutants [69]. However, biotinylation experiments revealed an increase in total and surface expressions of the core glycosylated form of p.T873I mutant, whereas p.L1075P exhibited a significant surface expression increase.

Later, Gualandi et al. have identified a double heterozygosity for pathogenic variants in *SCN5A* and *TRPM4* genes in a BrS patient [70]. The segregation analysis in the proband's parents revealed a paternal origin of the *SCN5A*-p.L1501V variant, while the *TRPM4*-p.G844D variant is inherited from the mother. The first variant was associated with Brugada and congenital LQT syndromes, while the second one is deemed linked to cardiac conduction disorders [23]. On the molecular level, the functional characterization of TRPM4-p.G844D mutant revealed an increased current density related to an impaired endocytosis and deregulation of SUMOylation process [23]. On the other hand, the clinical history of the present family did not show any major arrhythmogenic events or sudden cardiac death. This suggests that the presence of the two variants in both *SCN5A* and *TRPM4* genes is required for the full clinical expression of BrS, which is consistent with a digenic inheritance [70].

More recently, the molecular screening of 19 BrS-related genes was performed on a 64-year-old man presented with isolated exertional dyspnea, hypertension, chronic kidney disease, coronary disease, and a type 1 Brugada ECG pattern. The affected patient was a carrier of two TRPM4 null alleles (IVS9+1G>A and p.W525X) resulting in the absence of functional hTRPM4 proteins [21].

### 3.3. Pathophysiological Mechanisms Leading to Brugada Syndrome and Cardiac Conduction Diseases

The pathophysiological role of TRPM4 in cardiac conduction disease and BrS remains unclear. It is paradoxical that both TRPM4 gain- and loss-of-function are associated with similar phenotypes. It was proposed that in analogy to the phenomenon of supernormal excitability and conduction [71], both gain- and loss-of-function variants of TRPM4 channels may slow the conduction by affecting the availability of the cardiac sodium channels Na_v1.5_. TRPM4 gain-of-function may inactivate the sodium channels via the depolarization of the resting membrane potential, while a loss-of-function could induce a hyperpolarization of the membrane potential, leading to a reduced cellular excitability and conduction [42, 69]. These hypotheses were partially supported by some recent *in vivo* studies [18, 40, 72]. Regarding TRPM4 loss-of-function, the invalidation of the *TRPM4* gene in mice induced a multilevel conduction block without any modification of the ventricular AP characteristics [40]. Different results were obtained by Mathar and colleagues [18], illustrating no differences in the standard ECG parameters. Meanwhile, both studies did not report any modification of the membrane resting potentials. In addition, no reduction of the sodium current was observed in TRPM4^−/−^ mice compared to TRPM4^+/+^. As regards the TRPM4 gain-of-function, Pironet and colleagues have overexpressed wild-type TRPM4 channels in living mice via tail vein injection of AAV9 particles. The authors did not observe any increase in conduction abnormalities in mice overexpressing TRPM4 [72].

Altogether, these findings plead in favor of the implication of multiple factors rather than simple TRPM4 gain- or loss-of-function. These might include environmental factors, cell-specific effects, or the interaction between TRPM4 and interacting partners.

### 3.4. Congenital Long QT Syndrome

**Figure 3:**
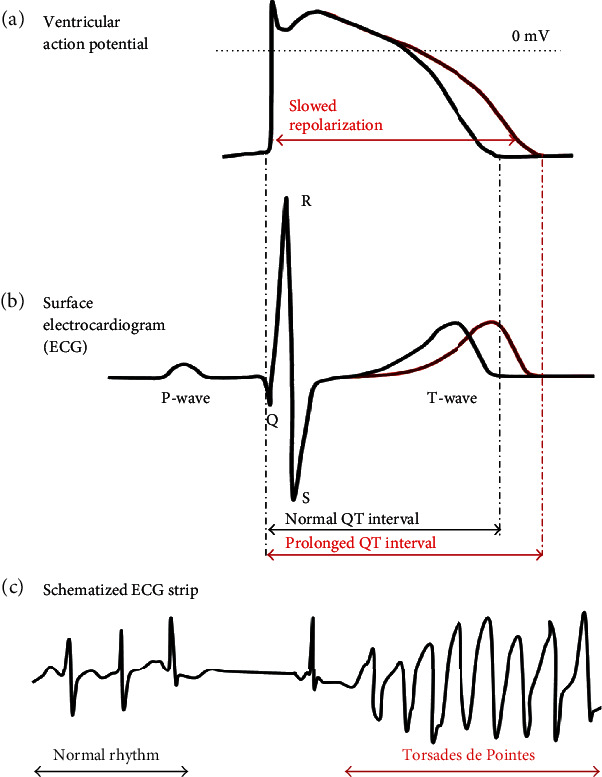
Schematic representation of ventricular action potentials and related ECG signals. (a, b) Normal and prolonged ventricular action potential and it related QT interval. (c) Schematic representation of ECG recording presenting the onset of Torsades de Pointes in a patient with long QT syndrome. Adapted from [77].

Congenital long QT syndrome (LQTS) is the most common inherited cardiac channelopathies. It is characterized by an impaired ventricular repolarization, which prolongs the heart rate-corrected QT interval (QTc) leading to an increased susceptibility to “Torsades de Pointes” and sudden cardiac death (Figure 3). The clinical diagnosis of LQTS is based on the combination of the medical and familial history and the 12-lead ECG of the patients [73–75]. According to the European Society of Cardiology guidelines, LQTS diagnosis can be made in case the QTc is more than 460 ms and the patient presents some antecedents, mainly a family history of SCD and an unexplained syncope. However, in asymptomatic patients without a family history of the disease, a QTc > 480 ms is required for the diagnosis of LQTS [76].

So far, mutations in 17 genes have been associated with LQTS [75, 78]: (1) three genes were classified as definitive genes for typical LQTS (*KCNQ1*, *KCNH2*, and *SCN5A*), (2) four genes with a strong evidence for causality in LQTS with atypical features (*CALM1*, *CALM2*, *CALM3*, and *TRDN*), (3) one gene with a moderate level evidence for causing this disease (CACNA1C), and (4) nine genes as having limited or disputed evidence as LQTS-causative genes (*AKAP9*, *ANK2*, *CAV3*, *KCNE1*, *KCNE2*, *KCNJ2*, *KCNJ5*, *SCN4B*, and *SNTA1*) [78].

Recently, Hof and colleagues have identified four TRPM4 variants through the screening of a cohort of 178 LQTS patients with no mutations in the major LQTS genes. Two of these variants, p.V441M and p.R449W, were characterized using the whole-cell configuration of the patch clamp technique [20]. Both variants showed a decreased density of TRPM4 current. However, no further investigations were performed to understand the cellular mechanism underlying the observed loss-of-function [20]. In addition, the contribution of these variants to the prevalence of cardiac arrhythmogenic disease remains unveiled [20].

## 4. Conclusions

In the heart, several studies highlighted the central role of TRPM4 in the cardiac conduction. In this sense, many pathogenic variants of the TRPM4 gene have been associated with different forms of conduction defects. Other variants were identified in BrS and LQTS patients. These variants were associated with either gain- or loss-of-function of TRPM4 channels for a similar clinical phenotype. These findings plead in favor of the implication of multiple factors rather than simple TRPM4 gain- or loss-of-function. Thus, further investigations are needed to understand the pathophysiological mechanisms underlying arrhythmias and conduction failures associated with TRPM4 dysfunction.

In addition to the described studies, TRPM4 channels were implicated in other cases of unexpected sudden natural death and cardiovascular disorders [79–83]. As such, TRPM4 channels may represent an attractive therapeutic target.

## Figures and Tables

**Table 1:** List of genetic variants of TRPM4 found in patients displaying cardiac conduction disorders.

Variants	Protein substitution	Diseases	Effect	Ref.
c.19G>A	p.E7K	PFHB1	G.F	[41]
c.301G>A	p.A101T	CHB	L.F	[49]
c.393G>C	p.Q131H	RBBB	n.d	[50]
c.490C>T	p.R164W	ICCD	G.F	[23]
c.878A>G	p.Q293R	AVB	n.d	[50]
c.1127T>C	p.I376T	PFHB1	G.F	[19]
c.1294G>A	p.A432T	ICCD (RBBB)	G.F/L.F	[23], [47], [48]
c.1744G>A	p.G582S	RBBB/AVB	G.F	[47]
c.1294G>A; c.1744G>A	p.A432T-p.G582S	AVB	L.E	[47]
c.2368T>C	p.Y790H	AVB	n.d	[50]
c.2531G>A	p.G844D	ICCD	G.F	[23]
c.2561A>G	p.Q854R	CHB	G.F	[49]
c.2741A>G	p.K914X	AVB	n.d	[50]
c.2908C>T	p.P970S	RBBB	n.d	[50]
c.3130A>T	p.S1044C	CHB	L.F	[49]
c.301G>A; c.3611C>T	p.A101T-P1204L	CHB	L.F	[49]

PFHB1: progressive familial heart block type I; RBBB: right bundle branch block; ICCD: isolated cardiac conduction disease; AVB: atrioventricular block; CHB: complete heart block; G.F: gain of function; L.F: loss of function; L.E: loss of expression.
